# Long Noncoding RNA H19: A Novel Therapeutic Target Emerging in Oncology *Via* Regulating Oncogenic Signaling Pathways

**DOI:** 10.3389/fcell.2021.796740

**Published:** 2021-12-16

**Authors:** Baokang Wu, Yizhou Zhang, Yang Yu, Chongli Zhong, Qi Lang, Zhiyun Liang, Chao Lv, Feng Xu, Yu Tian

**Affiliations:** ^1^ Department of General Surgery, Shengjing Hospital of China Medical University, Shenyang, China; ^2^ Department of Surgery, Jinzhou Medical University, Jinzhou, China

**Keywords:** H19, oncogene, signaling pathway, therapeutic target, cancer

## Abstract

Long noncoding RNA H19 (H19) is an imprinting gene with only maternal expression that is involved in regulating different processes in various types of cells. Previous studies have shown that abnormal H19 expression is involved in many pathological processes, such as cancer, mainly through sponging miRNAs, interacting with proteins, or regulating epigenetic modifications. Accumulating evidence has shown that several oncogenic signaling pathways lead to carcinogenesis. Recently, the regulatory relationship between H19 and oncogenic signaling pathways in various types of cancer has been of great interest to many researchers. In this review, we discussed the key roles of H19 in cancer development and progression *via* its regulatory function in several oncogenic signaling pathways, such as PI3K/Akt, canonical Wnt/β-catenin, canonical NF-κB, MAPK, JAK/STAT and apoptosis. These oncogenic signaling pathways regulated by H19 are involved in cell proliferation, proliferation, migration and invasion, angiogenesis, and apoptosis of various cancer cells. This review suggests that H19 may be a novel therapeutic target for cancers treatment by regulating oncogenic signaling pathways.

## 1 Introduction

In human genomic, only 2% of all human genes are protein coding genes, while the 98% genes are transcribed into non-coding RNA (ncRNA). According to the length of nucleotide sequences, ncRNA can be divided into short ncRNA and long ncRNA (lncRNA). LncRNA with transcript length of >200 nucleotides (He et al., 2014). Recently, increasing evidence has uncovered the importance of lncRNA, they could participate in gene expression by regulating epigenetic, transcriptional, and posttranscriptional levels (Charles Richard and Eichhorn, 2018). H19 was the first discovered lncRNA and has a total length of 2.3 kb (Brannan et al., 1990); it is encoded by a conserved gene cluster H19 locus located on chromosome 11p15.5 in humans and on chromosome seven in mice (Monnier et al., 2013). Similar to mRNAs, H19 has both exons and introns (containing five exons and four introns) (Ghafouri-Fard et al., 2020). H19 is an imprinting gene with only maternal expression. IGF2 is a reciprocally imprinted gene of H19 and only paternal expression, which is located 90 kb upstream of the H19 gene (Thorvaldsen et al., 1998). Generally, H19 is highly expressed in most tissues of the early stages of embryogenesis or placenta, while significantly declined postnatally, except for several adult tissues, such as skeletal muscle, cartilage, and cardiac muscle (Choong et al., 2019; Wang J. et al., 2020). Although, H19 RNA molecules can be detected in the cytoplasm and nucleus, H19 RNA is mainly present in the cytoplasm and functions by regulating RNA or ribosomes (Schoenfelder et al., 2007). Under normal conditions, the expression of H19 can be regulated by an epigenetic mechanism. When the imprinting control region (ICR) on the paternal chromosome located upstream of H19 is hypermethylated, which inhibited H19 expression and activated IGF2 expression; while hypomethylation of ICR on the maternal chromosome leads to contrary results. Enhancer regions located downstream of H19 can activate IGF2 expression when the paternal allele ICR region is hypermethylated, while activating H19 expression when the maternal allele ICR region is hypomethylated. Histone deacetylases inhibit H19 expression by interacting with CpG binding protein two to bind to methylated CpG dinucleotides (Yang Z. et al., 2021). Additionally, increasing evidence has reported that the expression of H19 can be regulated by transcription factors, including forkhead Box A1, forkhead Box F2, hypoxia-inducible Factor 1 subunit alpha, paxillin, E2F transcription Factor 1, SRY-sex determining region Y-Box 2, and paternally expressed gene 3 (Berteaux et al., 2005; Marášek et al., 2015; Ye et al., 2016; Wu W. et al., 2017; Zhang J. et al., 2019; Xu et al., 2019; Yang Z. et al., 2021).

H19 plays a key role in regulating different processes in cells. Firstly, H19 functions as a competitive endogenous RNA (ceRNA) (Pope et al., 2017), and ceRNA is referred to as a regulatory network that regulates mRNA expression through “sponging” target miRNA (lncRNA/pseudogene-miRNA-mRNA) (Su et al., 2021). MiR-675, one of the targeted miRNAs of H19, is embedded in H19’s first exon, and H19 has been shown to inhibit the growth of the placenta before birth by regulating the processing of miR-675. H19 can also interact with other miRNAs, such as miR-326 (Wei et al., 2019), miR-29a (Cheng et al., 2019), miR-124-3p (Liu et al., 2019), miR-152-3p (Zheng et al., 2020), miR-22-3p (Gan et al., 2019), miR-29b-3p (Zhong et al., 2021), miR-193a-3p (Ma et al., 2018), miR-612 (Yu et al., 2020), to regulate biological processes in various types of cells by modulating the expression of downstream target factors, including twist family bHLH transcription factor 1 (TWIST1), thymine DNA glycosylase, integrin β3 (ITGB3), bromodomain containing protein 4 (BRD4), Snail1, high mobility group box 1 (HMGB1), presenilin-1 (PSEN1), and Bcl-2. Secondly, H19 is involved in regulating different processes in various types of cells by interacting with different proteins, such as methyl-CpG-binding domain protein 1 (MBD1) (Monnier et al., 2013; Zhang et al., 2017), polypyrimidine tract-binding protein 1 (PTBP1) (Liu et al., 2018), enhancer of zeste homolog 2 (EZH2) (Luo et al., 2013), IGF2 mRNA-binding protein 1 (IGF2BP1) (Runge et al., 2000), p53 (Yang et al., 2012), K homology-type splicing regulatory protein (KSRP) (Giovarelli et al., 2014), zinc finger E-box-binding homeobox 1 (ZEB1) (Song et al., 2017). Thirdly, H19 can participate in epigenetic regulation to regulate gene expression by recruiting epigenetic regulatory factors involved in histone methylation (Alipoor et al., 2020).

**FIGURE 1:**
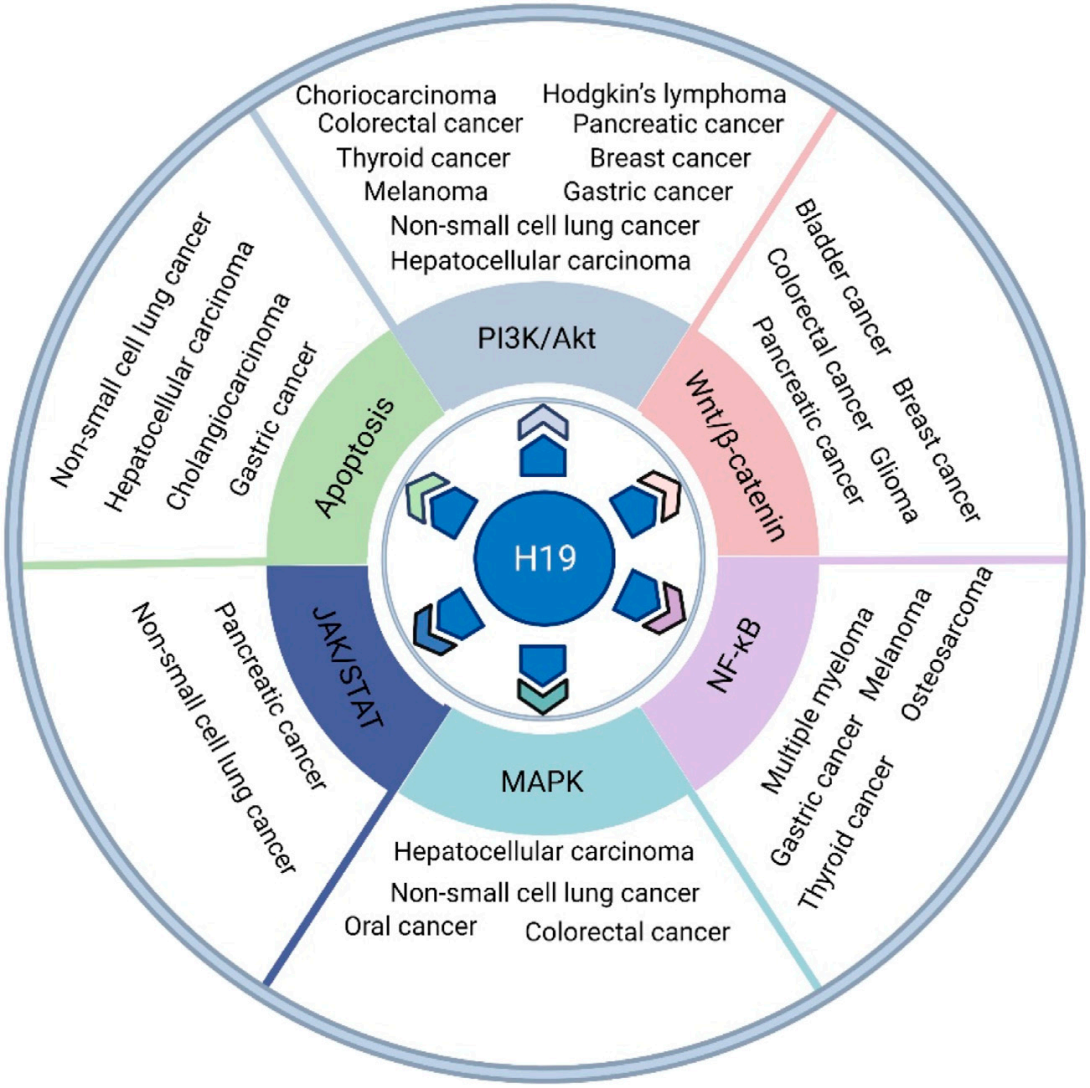
The role of H19 in various types of cancer, such as gastric cancer, HCC, pancreatic cancer, CRC, breast cancer, thyroid cancer, NSCLC, melanoma, Hodgkin’s lymphoma, choriocarcinoma, glioma, bladder cancer, osteosarcoma, multiple myeloma, oral cancer and cholangiocarcinoma, *via* its regulatory function in several oncogenic signaling pathways, such as the PI3K/Akt, canonical Wnt/β-catenin, canonical NF-κB, MAPK, JAK/STAT and apoptosis pathways.

Under pathological processes, such as cancer, H19 can be re-expressed. There is controversy about whether H19 acts as a tumor suppressor gene or oncogenic factor (Gibb et al., 2011). H19 plays differential roles in regulating biological processes in a variety of different types of cancer cells. Abnormal signaling pathways are closely associated with the processes of cancers. In this review, we provide important clues for understanding the key roles of the H19 functional network in signaling pathways and identifying new therapeutic targets for several cancers (Figure 1), such as gastric cancer, hepatocellular carcinoma (HCC), pancreatic cancer, colorectal cancer (CRC), breast cancer, thyroid cancer, non-small cell lung cancer (NSCLC), melanoma, Hodgkin’s lymphoma, choriocarcinoma, glioma, bladder cancer, osteosarcoma, multiple myeloma (MM), and oral and cholangiocarcinoma.

## 2 Oncogenic Signaling Pathways Regulated by H19 in Cancer

### 2.1 PI3K/Akt Signaling Pathway

Increasing evidence has shown the emerging roles of the phosphatidylinositol 3-kinase (PI3K)/Protein kinase B (Akt) signaling pathway in cell proliferation, differentiation, migration, angiogenesis, apoptosis, and other physiological activities (Teng et al., 2021). Under abnormal conditions, this signaling pathway can be overactivated, and PI3K can activate its downstream factor serine/threonine kinase Akt to regulate gene expression. Recently, a large number of studies have reported a positive correlation between H19 and the PI3K/Akt pathway in various types of cancer, suggesting a promising therapeutic target for cancer treatment.

#### Gastric Cancer

According to GLOBOCAN 2018, 1,033,701 new cases and 782,685 deaths occur annually and are associated with gastric cancer, making it the sixth most common cancer and the second most common cause of death by cancer among 36 cancers (Bray et al., 2018), which has caused a significant burden worldwide. H19 is considered an oncogenic lncRNA that activates oncogenic PI3K/Akt signaling in gastric cancer. Based on a new database visualization website of GEPIA with TCGA data and GSE2685 and GSE13861 microarray data from the Gene Expression Omnibus (GEO) databases, H19 is highly expressed in gastric cancer tissue samples. From data from The Cancer Genome Atlas (TCGA) databases, high H19 expression gastric cancer patients had a shorter overall survival. Likewise, gastric cancer patients with high H19 expression had a shorter recurrence-free survival in GSE26253 microarray data from GEO databases. Both these suggest that H19 could be a prognostic value lncRNA in gastric cancer. Additionally, studies showed the same results. Researchers examined H19 expression levels at the *in vitro* cell level and found that H19 was upregulated in the gastric cancer cell lines AGS, MKN-45, and SGC-7901 compared with the normal gastric mucosa cell line GES-1. Moreover, researchers examined H19 expression levels in 12 pairs of fresh gastric cancer tissues and corresponding normal adjacent tissues, and the RT–qPCR results indicated that H19 was more highly expressed in gastric cancer tissues than in corresponding normal adjacent tissues (Sun et al., 2021). H19 positively correlates with miR-675 expression in gastric cancer. Runt domain transcription Factor 1 (RUNX1), an important tumor suppressor, is an important downstream molecule of the H19/miR-675 axis, and both H19 and miR-675 decrease RUNX1 expression. In AGS cells, the H19/miR-675 axis could enhance the activation of AKT/mTOR signaling by promoting the phosphorylation of Akt and mTOR (Liu et al., 2016). In AGS cells cotransfected with the RUNX1 overexpression plasmid and miR-675 mimic, overexpression of RUNX1 inhibited miR-675 mimic-induced Akt and mTOR miR-675 mimic phosphorylation. Taken together, these data demonstrated that H19-derived miR-675 regulated gastric cancer cell progression due to its ability to activate the Akt/mTOR pathway by inhibiting the downregulation of RUNX1 (Liu et al., 2016).

#### Hepatocellular Carcinoma

Liver cancer was the seventh most common malignancy (841,080 new cases, 4.7% of the total) and the third leading cause of cancer-related death among 36 cancers (781,631 deaths, 8.2% of the total) in 2018 (Bray et al., 2018). It is universally acknowledged that the pathogenesis of HCC is complex, genomics mechanisms play a vital role. To ease this burden, the genomics mechanisms of HCC need to be investigated. H19 has been observed to modulate the PI3K/Akt signaling pathway in HCC. In the HCC cell lines of Huh-7, MHCC-97H and HepG2, the relative RNA levels of both H19 and miR-675 detected by qRT–PCR were higher than those in normal human hepatic LO2 cells. After treatment with a miR-675 inhibitor and H19 siRNA, the qRT–PCR results showed that H19 and miR-675 levels were decreased, which enhanced the migration and invasion of HCC, accompanied by dramatically increased expression levels of Akt and Cdc25A and decreased expression levels of glycogen synthase kinase 3β (GSK-3β) (Lv et al., 2014). Taken together, these data demonstrated that downregulation of H19 and miR-675 expression can promote the migration and invasion of HCC cells *via* the Akt/GSK-3β/Cdc25A signaling pathway.

#### Pancreatic Cancer

Pancreatic cancer is one of the leading causes of human cancer-related death worldwide with increasing incidence, and there are more than 458,918 new cases and 432,242 deaths annually (Bray et al., 2018). It is very malignant with a high mortality rate due to the lack of specific therapies, and its pathogenesis remains unclear. Recently, H19 was determined to be a promising therapeutic target for pancreatic cancer. In an established nude mouse xenograft model using T3M4, PANC-1, COLO357 and CAPAN-1 cells with a siH19 mixture or lenti-H19-GFP, H19 overexpression could increase tumor volume and weight and promote tumor growth quickly, while H19 knockout exerted the opposite consequence (Ma et al., 2016). Neurosecretory protein (VGF) is a highly conserved inducible gene for neurotrophic factors and is associated with pancreatic neuroendocrine functions. H19 regulated the VGF expression level, which promoted pancreatic neuroendocrine neoplasm cell proliferation, migration and invasion. In QGP-1 cells, western blotting results showed that H19 could activate VGF-mediated phosphorylation of PI3K, Akt, and cAMP-response element binding protein (CREB). In the xenografted tumors, western blotting analysis identified similar results. In summary, these results demonstrated that H19 could promote pancreatic neuroendocrine neoplasm progression *via* the VGF-mediated PI3K/Akt/CREB pathway (Ji et al., 2019).

#### Colorectal Cancer

CRC ranks as the fourth most common malignancy (1,096,601 new cases, 6.1% of the total) in the world and was the fifth leading cause of cancer-related death (551.269 deaths, 5.8% of the total) in 2018 (Bray et al., 2018). The molecular pathogenesis mechanism of CRC is complex. A recent study focused on H19 playing a vital regulatory function in the progression of CRC *via* the PI3K/Akt signaling pathway, which provides a promising method for improving early diagnosis, predicting prognosis, and developing effective therapies. Researchers investigated the RNA expression data of CRC patients obtained from 622 CRC cancer tissues and 51 adjacent nontumor normal tissues from the TCGA data portal, and the results showed that H19 is highly expressed in human CRC tissues (Zhong et al., 2019). As mentioned above, H19 functions as a ceRNA to modulate the various processes of cells. Zhong and others constructed the ceRNA network of CRC using 10 lncRNAs, 5 pseudogenes, 122 mRNAs, and 39 miRNAs and found that H19 could sponge to target 6 miRNAs and interact with 38 mRNAs in this ceRNA; These CRC-related mRNAs participate in the PI3K/Akt signaling pathway. Western blot analysis revealed that H19 knockdown suppressed the expression of MET, ZEB1, and collagen type I alpha 1 (COL1A1) in the H19 ceRNA network mentioned above in both HT-29 and HCT116 cells. Additionally, overexpression of H19 is associated with poor prognosis and clinical parameters such as tumor grade, lymphatic invasion, metastasis, and TNM stage (Zhong et al., 2019).

#### Breast Cancer

Breast cancer is a globally observed malignancy (2,088,849 new cases, 11.6% of the total) and is the fourth leading cause of cancer-related death (626,679 deaths, 5.8% of the total) worldwide (Bray et al., 2018). Chemotherapeutics have been widely used for breast cancer treatment, either as single agents or in combination with other chemotherapeutic agents; however, the emergence of chemotherapeutic resistance has become a huge obstacle for breast cancer treatment (Du et al., 2021). Previous studies have shown that H19 is associated with the pathogenesis of breast cancer. Compared to healthy tissues, H19 is upregulated in approximately 73% of breast cancer tissues. Interestingly, a recent study demonstrated that H19 is associated with chemotherapeutic resistance in breast cancer *via* the PI3K/Akt signaling pathway (Han et al., 2018). Triple-negative breast cancer (TNBC), one of the subtypes of breast cancer, has a high metastatic capability and poor prognosis when compared to other subtypes (Guney Eskiler et al., 2018). Recently, Han and others found that H19 is significantly overexpressed and induces paclitaxel resistance in TNBC cell lines. Furthermore, H19 knockout rescued paclitaxel resistance in the TNBC cell line by decreasing the expression of phosphorylated Akt (Ser473) and Bcl-2 and increasing the expression of Bax and cleaved caspase-3. These data demonstrated that H19 knockout rescued paclitaxel resistance in TNBC *via* the Akt-mediated apoptotic signaling pathway (Han et al., 2018). Additionally, established xenograft nude mice using MDA-MB-231/PTX-shH19 cells and MDA-MB-231/PTX-shNC cells showed the same results as above.

#### Thyroid Cancer

Thyroid cancer has a lower malignancy than the above cancers, ranking ninth in incidence (567,233 new cases) with a lower leading cause of cancer-related death (41,071 deaths). However, the incidence of thyroid cancer has been increasing in many countries since the early 1980s and is approximately 3 times higher in women than men (Bray et al., 2018). It is necessary to understand its pathogenesis for effective prevention, early diagnosis, and better prognosis. H19 is involved in the expression of members of the PI3K/Akt signaling pathway to regulate thyroid cancer progression. H19 is over six-fold overexpressed in thyroid cancer tissues compared with adjacent normal tissues. H19 knockout inhibited cell viability and induced apoptosis of thyroid cancer cells by prominently increasing the expression of Bax and caspase three and repressing the expression of Bcl-2. Moreover, the authors found that the PI3K/Akt signaling pathway is involved in the process by which H19 silencing inhibits thyroid cancer cell viability and induces apoptosis with significantly decreased phosphorylated PI3K and phosphorylated Akt (Li et al., 2019).

#### Non-Small Cell Lung Cancer

Global statistics in 2018 showed that lung cancer is the most common cancer among malignant tumors, and its morbidity and mortality are the highest in males and third most common form of cancer and second most common cause of cancer-related death in females (Bray et al., 2018). NSCLC accounts for a high proportion of lung cancer cases. Epidermal growth factor receptor (EGFR) tyrosine kinase inhibitors, such as erlotinib, have been proven to benefit NSCLC by inhibiting the activation of EGFR gene mutations (Paez et al., 2004). However, drug resistance appears after approximately 12 months of drug use, which is a major challenge for cancer treatment. Increasing evidence has demonstrated that H19 is associated with drug resistance in lung cancer by modulating the PI3K/Akt signaling pathway. Chen and others found that H19 is downregulated in erlotinib-resistant EGFR mutant human NSCLC cell lines HCC827 and PC9. H19 downregulation confers erlotinib resistance. Moreover, H19 knockdown reduces the inhibitory effect of erlotinib on the phosphorylation of Akt. Increased AKT activation leads to erlotinib resistance in NSCLC. Researchers have also confirmed the above results in xenograft tumors (Chen et al., 2020).

#### Melanoma

Melanoma is a lethal form of skin cancer that originates from cells containing pigment (Hodis et al., 2012). The incidence of melanoma has continued to increase worldwide, and there were 287,723 new cases and 60,712 deaths in 2018 (Bray et al., 2018). The 5-years survival rate is very low when metastasis occurs. Recently, studies have demonstrated that the level of H19 expression is correlated with the migration and invasion of melanoma cells by regulating the activation of the PI3K/Akt signaling pathway (Zhu et al., 2018; Bi et al., 2019). Zhu and colleagues showed that H19 promotes the proliferation, invasion, and growth of melanoma cell line A375 by upregulating Akt phosphorylation, increasing the expression of matrix metalloproteinases 2 (MMP2), MMP9, and Slug and downregulating the expression of E-cadherin (Zhu et al., 2018). Schizandrin A (SchA), a novel antioxidant compound, has been proven to exert antitumor properties in melanoma. Bi and colleagues demonstrated that SchA is negatively correlated with the level of H19. SchA inhibited cell proliferation and migration and increased cell apoptosis in the melanoma cell line A375 by downregulating the expression of H19, which in turn inactivated the PI3K/Akt signaling pathway by decreasing the phosphorylation of PI3K and Akt. Furthermore, H19 overexpression exerted the opposite results (Bi et al., 2019). Therefore, H19 is a potential target for the treatment of melanoma.

#### Hodgkin’s Lymphoma

Hodgkin’s lymphoma is a lymphoid hematopoietic system malignant tumor with a gradually increasing incidence. In 2018, 287,723 new cases and 60,712 deaths occurred worldwide (Bray et al., 2018). Its pathogenesis is complex, and we need to further explore the potential mechanism of Hodgkin’s lymphoma. Recently, H19 was reported to be involved in the pathogenesis of Hodgkin’s lymphoma *via* the PI3K/Akt signaling pathway, which provides a novel strategy for this cancer treatment by regulating the expression of H19. Wang and colleagues detected tissues from 60 Hodgkin’s lymphoma patients and 40 reactive hyperplasia of lymph nodes. qRT–PCR results showed that H19 is significantly overexpressed in Hodgkin’s lymphoma tissues compared to reactive hyperplasia of lymph nodes. More importantly, overexpression of H19 promoted Hodgkin’s lymphoma cell proliferation by regulating Akt expression, while H19 knockout exerted the opposite results (Wang Y. et al., 2019).

#### Choriocarcinoma

Choriocarcinoma is a highly malignant trophoblastic cell tumor (Bazot et al., 2004). Although the incidence is relatively low, it easily metastasizes and has a poor prognosis. Thus far, chemotherapy has been the major treatment. It is necessary for us to understand the molecular mechanisms of choriocarcinoma to inhibit metastatic ability. H19 has been proven to be involved in the development of the human choriocarcinoma cell line JAR by acting on the PI3K/Akt signaling pathway. Yu and colleagues showed that H19 is related to the drug resistance mechanism of choriocarcinoma. H19 is significantly overexpressed in the methotrexate- and 5-fluorouracil-resistant human choriocarcinoma cell line JEG-3. H19 knockout inhibited the proliferation, migration and invasion and promoted the apoptosis of choriocarcinoma-resistant cells. More importantly, the authors also found that the PI3K/Akt/mechanistic target of rapamycin (mTOR) signaling pathway might be involved in H19-mediated effects. Western blot analysis showed that the levels of phosphorylated PI3K, Akt, and mTOR were dramatically increased in H19 knockout methotrexate- and 5-fluorouracil-resistant JEG-3 cells. Interestingly, after inhibition of phosphorylated PI3K/Akt/mTOR, the effects mediated by H19 were markedly reversed (Yu et al., 2019).

### 2.2 Canonical Wnt/β-Catenin Signaling Pathway

Increasing evidence has demonstrated that Wnt signaling pathways are involved in diverse physiological processes. Because Wnt proteins interact with the downstream factor β-catenin in the cascade reaction, the Wnt signaling pathways can be divided into two subpathways: canonical Wnt/β-catenin signaling and noncanonical Wnt/PCP and Wnt/Ca^2+^ signaling (Xu et al., 2020). At present, the canonical Wnt/β-catenin signaling pathway is the best-characterized pathway, and studies have shown that abnormal Wnt/β-catenin signaling contributes to the pathogenesis of various cancers (Zhang and Wang, 2020). Under pathogenic conditions, Wnt/β-catenin signaling is abnormally activated by the binding of Wnt ligands to the low-density lipoprotein receptor-related proteins 5/6 (LRP5/6) receptor and Frizzled receptor. Subsequently, the Frizzled receptor recruited disheveled (DVL), leading to LRP5/6 phosphorylation and the recruitment of Axin (Bilic et al., 2007). Furthermore, activated disheveled (DVL) inhibited the degradation of β-catenin mediated by GSK-3β and casein kinase 1α (CK1α) destruction complex, leading to an elevation of β-catenin levels in the cytoplasm. Elevation of cytosolic β-catenin could translocate into the nucleus to regulate Wnt target gene expression by interacting with either p300 or CREB-binding protein as a transcriptional coactivator for T cell-specific factor (TCF)/lymphoid enhancer-binding factor (LEF) (Bilic et al., 2007; Hu et al., 2020). Recently, increasing evidence has indicated that H19 expression is significantly associated with abnormal Wnt/β-catenin signaling pathways in various types of human cancers, such as pancreatic cancer, CRC, glioma, breast cancer, and bladder cancer. The molecular mechanisms are complex and are described in detail below.

#### Pancreatic Cancer

Abnormal expression of H19 and its downstream factors has been observed in pancreatic cancer by regulating the Wnt/β-catenin signaling pathway (Sun et al., 2019). PFTK1 (cyclin-dependent kinase 14, CDK14) mediates the phosphorylation of LRP5/6 involved in the Wnt/β-catenin pathway (Davidson et al., 2009). In 45 paired pancreatic ductal adenocarcinoma and noncancerous tissue samples, both H19 and PFTK1 were highly expressed and correlated with distant metastasis, advanced TNM stages, and overall survival. MiR-194 has been found to directly bind to upstream H19 and target downstream PFTK1 by directly binding to the 3′-untranslated region (3′-UTR). This H19/miR-194/PFTK1 axis modulated pancreatic ductal adenocarcinoma cell proliferation and migration through Wnt/β-catenin signaling by regulating the levels of phosphorylated LRP6 Snail and increasing phosphorylated β-catenin protein levels (Sun et al., 2019).

#### Colorectal Cancer

In CRC, H19 was found to be abnormally expressed and associated with the abnormally activated Wnt/β-catenin signaling pathway (Wu K.-f. et al., 2017; Ding D. et al., 2018). According to the TCGA database, H19 was significantly highly expressed in CRC tissues in comparison to normal tissues, while miR-29b-3p was significantly downregulated in the collected CRC tissues compared with adjacent normal tissues. Consistent with TCGA data, the same results were found in CRC patients and cell lines. H19 directly targeted miR-29b-3p to inhibit its expression. Moreover, miR-29b-3p directly targets downstream progranulin to participate in the activation of the Wnt/β-catenin signaling pathway, causing apparent alterations in the epithelial mesenchymal transition (EMT) process and remarkably different transcriptional activities of the β-catenin/T cell-specific factor (TCF) reporter plasmid (Ding D. et al., 2018). In summary, the H19/miR-29b-3p/progranulin axis is involved in the EMT process of CRC by regulating the Wnt/β-catenin signaling pathway. Additionally, H19 mediated drug resistance in CRC by regulating the Wnt/β-catenin signaling pathway. In the methotrexate-resistant colorectal cell line HT-29 (HT-29-R), H19 was significantly upregulated. H19 knockdown could restore the sensitivity of HT-29-R cells to methotrexate resistance through the Wnt/β-catenin signaling pathway by inhibiting the expression of β-catenin (Wu K.-f. et al., 2017).

#### Breast Cancer

H19 could regulate breast cancer cell processes by acting on the Wnt/β-catenin signaling pathway. In the paclitaxel-resistant (PR) cell subline (MCF-7/PR), H19 was upregulated, and could as a ceRNA *via* competitively binding miR-340-3p, which regulates cell proliferation, migration, invasion and apoptosis (Yan et al., 2020). YWHAZ (tyrosine 3-monooxygenase/tryptophan 5-monooxygenase activation protein zeta) is a direct downstream target of miR-340-3p, H19 regulates the breast cell progression miR-340-3p/YWHAZ axis, and the H19/miR-340-3p/YWHAZ axis altered EMT phenotype in breast cancer metastasis and invasion by regulating β-catenin expression, accompanied with changed expression of E-cadherin, Slug, Snail, Vimentin, Cyclin D1, and c-Myc (Yan et al., 2020). Breast cancer stem-like cells (BrCSCs) have a strong self-renewal ability and are considered the greatest obstacle for breast cancer treatment. H19 stimulated the symmetric division of BrCSCs. Let-7c is a protective indicator, and Wnt signaling is excessively activated in breast cancer. H19, as a ceRNA, inhibits Let-7c expression through the estrogen receptor 1/Wnt cycle to induce BrCSC symmetric division (Wang M. et al., 2019).

#### Glioma

Glioma is a common and highly malignant tumor of the central nervous system with a poor prognosis, which is not sensitive to radiotherapy and chemotherapy. As far, the pathogenesis of glioma is complex and unclear (Deangelis, 2001). Hence, identifying molecular targets for the diagnosis and treatment of gliomas is important. Increasing evidence had shown that H19 regulated the development of glioma by regulating the Wnt/β-catenin signaling pathway (Guan et al., 2019; Zhou et al., 2020). H19 expression was higher in glioma tissues and cell lines with a poor prognosis. H19 knockdown inhibited glioma cell proliferation, invasion, and migration, arrested the glioma cell cycle and induced glioma cell apoptosis. H19 modulated the above glioma cell processes by regulating the activation of the Wnt/β-catenin signaling pathway (Guan et al., 2019). As mentioned above, H19 functions as a ceRNA to regulate cell processes. H19 had been found to directly target miR-342, decreasing its expression. Consistent with the above results, H19 was highly expressed in glioma and directly targets miR-342 to regulate Wnt5a/β-Catenin to modulate cell proliferation, migration, and angiogenesis. H19 silencing reversed glioma cell growth and metastasis by regulating the miR-342-mediated Wnt5a/β-Catenin signaling pathway with decreased levels of vascular endothelial growth factor A (VEGFA), MMP9, Wnt5a, and β-catenin (Zhou et al., 2020).

#### Bladder Cancer

According to GLOBOCAN 2018, over 549,000 new bladder cancer cases and 200,000 deaths were estimated globally (Bray et al., 2018). Given its aggressiveness, it is considered the major obstacle for bladder cancer treatment, and patients with aggressive bladder cancer often have a poor prognosis with a 50% 5-years survival (Kim et al., 2010). A recent study demonstrated that H19 plays a vital role in bladder cancer invasion and metastasis (Luo et al., 2013). H19 is significantly upregulated in most bladder cancer tissues and invasive bladder cancer cell lines. EZH2, as a critical component of polycomb repressive complex 2 (PRC2), is associated with cancer metastasis (Wang et al., 2013). Luo and colleagues found that H19 is associated with EZH2, which increases the activation of the Wnt/β-catenin signaling pathway, resulting in decreased expression of E-cad. Taken together, these data demonstrated that the association of H19 upregulation with EZH2 increased bladder cancer metastasis by inhibiting Wnt/β-catenin-mediated E-cad expression (Luo et al., 2013).

### 2.3 Canonical NF-κB Signaling Pathway

The canonical nuclear factor kappa B (NF-κB) signaling pathway, one of the NF-κB pathways, plays an important role in multiple physiological and pathological processes. It is well established that the pathway can be activated by various cytokines, such as tumor necrosis factor alpha (TNF-α), interleukin-1 (IL-1), and lipopolysaccharides (LPS) (Hoesel and Schmid, 2013; Taniguchi and Karin, 2018). The engagement of these cytokines with their receptor initially activates the downstream cascade molecule IκK complex, which consists of three subunits, IκKα, IκKβ, and IκKγ, resulting in the phosphorylation of IκBα at S32 and S36 and subsequent ubiquitination and proteasomal degradation. p50/p65 dimers released from the IκBα/p50/p65 complex after the degradation of IκBα translocate to the nucleus to regulate the expression of inflammation- and immune-related genes (Hoesel and Schmid, 2013). Increasing studies have demonstrated that H19 regulates canonical NF-κB signaling pathway activation in various types of human cancers, such as gastric cancer, thyroid cancer, melanoma, osteosarcoma, and multiple myeloma.

#### Gastric Cancer

H19 has been reported to be associated with altered expression of the components of the canonical NF-κB signaling pathway in gastric cancer. In *Helicobacter pylori* (HP)-infected gastric cancer tissues and cells, H19 is noteworthily highly expressed. Additionally, HP infection promotes gastric cancer cell viability, migration, invasion and the inflammatory response. HP infection promotes IκBα ubiquitination and proteasomal degradation and p65 translocation, and this ability is enhanced by overexpression of H19. H19 overexpression promotes HP-induced gastric cancer cell proliferation, migration, and invasion by altering the expression of IκBα, phosphorylated IκBα and p65 in the canonical NF-κB signaling pathway (Zhang Y. et al., 2019).

#### Thyroid Cancer

In thyroid cancer, H19 may be a suppressor gene, and its overexpression inhibits cell viability, migration, and invasion but promotes cell apoptosis. Meanwhile, H19 knockdown has demonstrated contrary results. Furthermore, H19 overexpression negatively regulates insulin receptor substrate I expression to modulate the above processes in SW579 and TPC1 thyroid cancer cells. Decreased insulin receptor substrate I regulates the PI3K/Akt signaling pathway and NF-kB signaling pathway by decreasing the levels of phosphorylated PI3K, phosphorylated Akt, phosphorylated p65, and phosphorylated IκBα in SW579 and TPC-1 thyroid cancer cells (Wang et al., 2017).

#### Melanoma

Likewise, H19 exerts an important role in melanoma by regulating the NF-kB signaling pathway. H19 is significantly more highly expressed in melanoma tumor tissue than in adjacent healthy tissue. Moreover, H19 knockdown inhibits melanoma cell proliferation, migration and invasion. H19 knockdown decreases the levels of p65 and p50 to inhibit the activation of the NF-κB signaling pathway by inactivating the PI3K/Akt signaling pathway (Liao et al., 2018). These data showed that H19 regulates melanoma cell processes by inactivating the PI3K/Akt-mediated NF-κB signaling pathway.

#### Osteosarcoma

Osteosarcoma is a relatively rare type of cancer; however, similar to other cancers, its pathogenesis is complex and certainly places a burden on children and young adults (Botter et al., 2014). Increasing evidence has shown that the H19 is involved in the development of osteosarcoma, and the mechanisms are more complex and need further investigation. H19 regulates osteosarcoma cell processes by acting on the NF-κB signaling pathway, and as one of the mechanisms, H19 has been investigated (Zhao and Ma, 2018). H19 was significantly overexpressed in osteosarcoma tumor tissue compared with adjacent healthy tissue. Moreover, H19 has been associated with distant metastasis of osteosarcoma and overall survival of patients with osteosarcoma. Osteosarcoma cell migration and invasion have been significantly reduced by H19 knockdown, which may be associated with inactivation of the NF-κB signaling pathway. H19 knockdown apparently increases the level of IκBα by regulating the activation of the PI3K/Akt signaling pathway in osteosarcoma cell lines compared with normal cells (Zhao and Ma, 2018).

#### Multiple Myeloma

MM is a bone marrow-resident hematological malignancy of plasma cells and is responsible for 159,985 cases worldwide (Bray et al., 2018; Minnie and Hill, 2020). Although the 5-years overall survival rate of MM has increased in recent decades, the incidence of MM has increased uniformly since 1990 (Cowan et al., 2018). Based on the increased incidence, it is urgent to well characterize the molecular mechanisms for effective and better treatment of MM. Recently, H19, as a cancer-promotive gene in MM that acts on the NF-κB signaling pathway, was investigated (Sun et al., 2017). H19 significantly overexpressed MM tissues and cells and was associated with a worse prognosis in patients with MM. Moreover, H19 knockdown inhibited the activation of the NF-κB signaling pathway by reducing the levels of phosphorylated IκBα and nuclear P65 to modulate MM cell growth. Additionally, a positive correlation between H19 and the levels of the cytokines IL-6 and IL-8 was observed in MM (Sun et al., 2017).

### 2.4 MAPK Signaling Pathway

The mitogen-activated protein kinase (MAPK) signaling pathways have been proven to regulate various cell processes and play an important role in carcinogenesis. There are three main classical MAPK subgroup pathways: extracellular regulated protein kinases (ERK)/MAPKs, c-Jun N-terminal kinase (JNK)/MAPKs, and p38/MAPKs (Peluso et al., 2019). Under pathogenic conditions, all three subgroup pathways are involved in carcinogenesis. In the ERK/MAPK pathway, the first line of cytosolic intermediate Ras is activated after growth factors engage with their receptors; subsequently, activated Ras activates the downstream Raf/MEK/ERK kinase cascade to inhibit cell apoptosis (Braicu et al., 2019). The JNK/MAPK and p38/MAPK pathways are activated by various stress stimuli to promote apoptosis. After various stress stimuli, the MKK/JNK kinase cascade and MKK/p38 kinase cascade are activated (Zeke et al., 2016; Anton et al., 2021). Among cancers, such as liver cancer, colon cancer, non-small cell lung cancer, and oral cancer, H19 is associated with the progression of these cancers by altering the level of the component of the MAPK signaling pathway.

#### Hepatocellular Carcinoma

H19 is overexpressed and results in a poor clinical outcome in patients with HCC. Of note, H19 overexpression is slightly associated with large tumors and portal vein tumor thrombi. H19 could regulate migration and invasion by inducing EMT in HepG2 cells. Furthermore, H19 functions as a ceRNA to hijack miR-193b and protect ERK2. The ceRNA network of H19/miR-193b/MAPK1 is abnormally activated in HCC, leading to the activation of the MAPK signaling pathway, which promotes aggressiveness in HCC (Ye et al., 2020). Similar to the above results, H19 is highly expressed in CD133 + HCC stem cells, and H19 overexpression activates the MAPK signaling pathway by increasing the levels of phosphorylated MAPK and phosphorylated ERK1/2. Additionally, H19 has a dramatic effect on oxidative stress. H19 knockdown also causes a significant enrichment of reactive oxygen species in CD133 + HCC stem cells (Ding K. et al., 2018).

#### Colorectal Cancer

As mentioned above, H19 promotes the migration and invasion of CRC cells by regulating PI3K/Akt. Recently, a study also investigated whether H19 may promote metastasis and invasion in CRC by activating the Ras/ERK signaling pathway. H19 could regulate the activation of RAS and subsequently affect the levels of phosphorylated Raf, phosphorylated MEK and phosphorylated ERK, resulting in CRC cell migration and invasion (Yang et al., 2018). Moreover, H19 regulates CRC migration and invasion through a heterogeneous nuclear ribonucleoprotein A2B1 (hnRNPA2B1)-dependent ERK/MAPK pathway with increased expression of Raf-1 (Zhang et al., 2020).

#### Non-Small Cell Lung Cancer

Similarly, H19 participates in the process of NSCLC metastasis by regulating the expression of components of the MAPK signaling pathway. H19 knockdown exerts a negative effect on the migration and invasion of NSCLC A549 cells by modulating MAPK signaling pathway-related protein ERK1/2 expression and other signaling pathway protein expressions associated with cell proliferation and cell adhesion, such as metastasis-associated in colon cancer 1 (MACC1), EGFR, and β-catenin (Wang et al., 2016).

#### Oral Cancer

Oral cancer is a common type of cancer worldwide, and the GLOBOCAN 2018 indicated that there were 354,864 new cases and 177,384 deaths from oral cancer in 2018 (Bray et al., 2018). This has aroused attention due to its gradually increasing incidence and mortality rate. Cancer-associated fibroblasts (CAFs) play a promoting role in cancer (Chen and Song, 2019). According to the TCGA database, bioinformatic analysis showed that H19 is a key long noncoding RNA in oral CAFs. H19 knockdown affects oral CAFs glycolysis, proliferation, and migration processes by regulating the miR-675-5p/PFKFB3 axis. The MAPK signaling pathway is well known to be involved in glycolysis pathways. H19 knockdown dramatically inhibits the MAPK signaling pathway by reducing the levels of ERK, phosphorylated ERK, phosphorylated p38, and JNK in oral CAFs. Consistent with the above results, H19 knockdown in CAFs exhibits an inhibitory effect on tumor growth (Yang J. et al., 2021).

### 2.5 JAK/STAT Signaling Pathway

The Janus kinase (JAK)/signal transducer and activator of the transcription (STAT) signaling pathway has been found to be a crucial oncogenic signaling pathway that participates in various physiological and pathological processes (Verhoeven et al., 2020). The major components of this pathway include cytokines and growth factors, cognate receptors, JAK family members (JAK1, JAK2, JAK3, and TYK2), and STAT family members (STAT1, STAT2, STAT3, STAT4, STAT5A, STAT5B, and STAT6). Cytokines and growth factors bind to their respective receptors and subsequently induce receptor dimerization, which results in JAK phosphorylation. Phosphorylated JAKs bind and phosphorylate the cytoplasmic tyrosine residues of the receptors, where they recruit STAT proteins. Then, STAT proteins are phosphorylated by JAKs, and phosphorylated STAT proteins form homodimers or heterodimers that translocate into the nucleus to regulate target gene expression (Gutiérrez-Hoya and Soto-Cruz, 2020). Suppressors of cytokine signaling (SOCS) proteins can inhibit the JAK/STAT signaling pathway by binding to the SH2 domains of JAKs, and protein inhibitors of activated STAT (PIAS) and protein tyrosine phosphatases (PTPs) can negatively regulate this pathway by inhibiting STAT-DNA binding and are involved in the suppression of various cytokine signals (Ou et al., 2021). The relationship between H19 and the JAK/STAT signaling pathway has been elucidated in several cancers, and the detailed mechanisms are described in the following sections.

#### Pancreatic Cancer

H19 is significantly highly expressed in pancreatic cancer cells and regulates the processes of the migration, invasion, EMT and chemoresistance of pancreatic cancer cells. The STAT3 pathway participates in the modulation of these processes. SCOS5, a member of the SCOS family, inhibits STAT3 activation, and H19 regulates the above effects *via* SOCS5/STAT3 signaling in pancreatic cancer cells. Interestingly, miR-675-3p, as a direct target of H19, regulates SCOS5 expression and STAT3 phosphorylation. Herein, the H19/miR-675-3p/SOCS5/STAT3 axis plays an important role in pancreatic cancer cells, which revealed potential targets for the treatment of pancreatic cancer (Wang F. et al., 2020).

#### Non-Small Cell Lung Cancer

H19 is overexpressed in NSCLC A549 and H1299 cells, while miR-17 is downregulated. Bioinformatics analysis based on ChipBase, LncRNAdb, and StarBase showed that H19 is negatively associated with miR-17 expression. H19 as a ceRNA directly targets miR-17. STAT3, an important member of the JAK/STAT signaling pathway, is a downstream target of miR-17 (Huang et al., 2018). In NSCLC cells, H19 regulates cell growth, migration, and invasion progression *via* the miR-17/STAT3 axis (Huang et al., 2018).

### 2.6 Apoptosis Signaling Pathway

Apoptosis is the classic form of programmed cell death, which plays a crucial role in the development of cancer. There are two classical apoptosis signaling pathways: the extrinsic pathway and the intrinsic pathway (Strasser et al., 1995). The interaction of Fas ligand (FasL), TNF- related apoptosis-inducing ligand (TRAIL), and TNF-α with their receptors induces the initiation of the extrinsic apoptotic pathway; consequently, Fas-associating protein with a novel death domain (FADD)-mediated caspase-8 activation activates caspase-8 to activate caspase-3, -6, and -7, which induce cell apoptosis. Additionally, caspase-8 processes truncated Bid and is then involved in the Bcl-2 family pathway (Ozyerli-Goknar and Bagci-Onder, 2021). The intrinsic pathway, also called the mitochondrial pathway, can activate cytokine deprivation, intracellular damage, and oncogenes; subsequently, proapoptotic members of the Bcl-2 family of proteins receive signals, leading to the release of cytochrome C from the mitochondria. The released cytochrome C triggers apoptotic protease activating factor 1 (APAF-1)-mediated activation of caspase-9, which leads to cell apoptosis by activating caspase-3, -6, and -7 (Strasser et al., 1995; Ozyerli-Goknar and Bagci-Onder, 2021). Increasing evidence has shown that H19, as an oncogene, induces cancer cell apoptosis.

#### Gastric Cancer

In gastric cancer, H19 is overexpressed and plays an important role in apoptosis. Overexpression of H19 promotes cell proliferation and inhibits cell apoptosis by directly targeting miR-675. FADD, as a target gene of miR-675, is involved in the effects mediated by the H19/miR-675 axis, and its expression is downregulated; subsequently, downregulation of FADD inhibits its downstream caspase-3 and caspase-8 expression (Yan et al., 2017).

#### Hepatocellular Carcinoma

The effect of H19 on the components of the apoptosis signaling pathway in HCC was investigated. In HBV-induced hepatoblastoma, H19 was overexpressed and directly targeted miR-675 to regulate FADD, caspase-8, and caspase-3 expression. Moreover, H19 upregulated protein tyrosine kinase 2 (PTK2) by targeting downstream miR-138. H19, as an oncogene, inhibited cell apoptosis in hepatoblastoma *via* both signaling axes (Ge et al., 2019b). Recently, H19 single nucleotide polymorphisms (SNPs) were proven to be associated with cell apoptosis in HCC. The T allele of the H19 rs217727 polymorphism dramatically increased the survival rate of patients with HCC. Furthermore, the T allele of the H19 rs217727 polymorphism suppressed caspase-3 and caspase-8 expression *via* miR-675/FADD to inhibit cell apoptosis (Ge et al., 2019a).

#### Non–Small Cell Lung Cancer

Similarly, H19 could regulate NSCLC apoptosis by altering the expression of apoptosis-related proteins. H19 and its target gene miR-675 were significantly upregulated in NSCLC tissues and cells. p53 was found to be directly downstream of miR-675, and its expression was inversely associated with H19 and miR-675 but positively associated with its downstream apoptosis-related proteins Bax/Bcl-2. Taken together, the H19/miR-675/p53 axis was found to inhibit cell apoptosis by regulating the expression of Bax and Bcl-2 (Zheng et al., 2019).

#### Cholangiocarcinoma

Cholangiocarcinoma is an aggressive malignant tumor originating in the extrahepatic bile duct (Banales et al., 2020). Currently, chemotherapy and radiation therapy are the main treatment therapies, but they have a poor prognosis and a shorter 5-years survival rate (7–20%) (Su et al., 2019; Banales et al., 2020). H19 has been proven to be associated with the pathogenesis of cholangiocarcinoma, which may provide a potential therapeutic target. Bioinformatic analysis based on the TCGA and GEO databases found that H19 is a hub gene in cholangiocarcinoma. H19 was found to be overexpressed under the effect of the transcription factor hypoxia-inducible Factor 1α (HIF1α). Additionally, H19 was found to promote Bcl-2 expression by downregulating miR-612 expression, which promoted cholangiocarcinoma (Yu et al., 2020).

## 3 Conclusion and Perspectives

**FIGURE 2:**
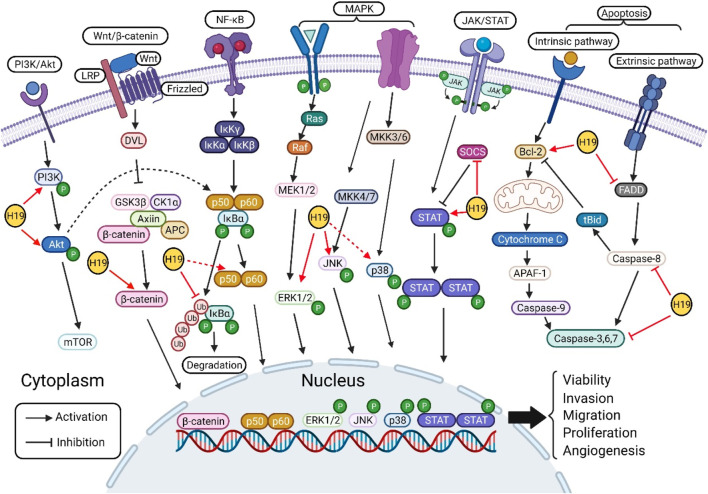
Molecular targets of H19 in various oncogenic signaling pathways. H19 regulates the phosphorylation of PI3K and Akt in PI3K/Akt signaling pathway. H19 targets the key component of canonical Wnt/β-catenin signaling pathway, increase the level of β-catenin. In NF-κB signaling pathway, H19 modulates the phosphorylation of IκBα, p65, and p50 to regulate cell processes. H19 activates the phosphorylation of ERK1/2, JNK, and p38 in MAPK signaling pathway. H19 activates the phosphorylation of STAT and inhibits the expression of SOCS in STAT signaling pathway. Furthermore, H19 regulates apoptosis signaling pathway by targeting Bcl-2, FADD, caspase-8, and caspase-3. Finally, H19 regulates cell proliferation, proliferation, migration and invasion, angiogenesis, drug resistance and apoptosis by directly/indirectly acting on the above key molecules of signaling pathways.

Since its discovery, an increasing number of studies have focused on the roles of H19 in the pathogenesis of various types of cancer through different mechanisms, such as sponge miRNAs, interactions with proteins, and epigenetic modifications. In this review, the role of H19 in the pathogenesis of cancers, such as gastric cancer, HCC, pancreatic cancer, CRC, breast cancer, thyroid cancer, NSCLC, melanoma, Hodgkin’s lymphoma, choriocarcinoma, glioma, bladder cancer, osteosarcoma, MM, oral cancer and cholangiocarcinoma, *via* its regulatory function in several oncogenic signaling pathways, such as the PI3K/Akt, canonical Wnt/β-catenin, canonical NF-κB, MAPK, JAK/STAT and apoptosis pathways, was analyzed. Furthermore, this review summarized the main regulatory effects of H19 on cell proliferation, proliferation, migration and invasion, angiogenesis, drug resistance and apoptosis of cancer by directly acting on key molecules or indirectly altering the levels of proteins associated with these signaling pathways (Figure 2) (Table 1). Based on the above results, H19 may be a promising therapeutic target/biomarker for the diagnosis, prevention, treatment and prognosis of different types of cancer.

**TABLE 1:** H19 exerts its regulatory function in several types of cancer *via* regulating several oncogenic signaling pathway.

Signaling pathway	Cancer type	Oncogene/suppressor gene	H19 expression	Molecule mechanism	Cell processes	References
PI3K/Akt	Gastric cancer	Oncogene	Overexpression	↑p-Akt, ↑p-mTOR, ↓RUNX1	Enhance proliferation and invasion	Liu et al. (2016)
	Hepatocellular carcinoma	Suppressor gene	Downregulation	↑Akt, ↑Cdc25A, ↓GSK-3β	Promote migration and invasion	Lv et al. (2014)
	Pancreatic cancer	Oncogene	Overexpression	↑VGF, ↑p-PI3K, ↑p-Akt, ↑p-CREB	Promote progression	Ji et al. (2019)
	Colorectal cancer	Oncogene	Knockdown	↓MET, ↓ZEB1, ↓COL1A1	Predict prognosis	Zhong et al. (2019)
	Breast cancer	Oncogene	Knockout	↑Bax, ↑cleaved caspase-3, ↓p-Akt, ↓Bcl-2	Rescue drug resistance	Han et al. (2018)
	Thyroid cancer	Oncogene	Knockdown	↑Bax, ↑caspase 3, ↓Bcl-2, ↓p-PI3K, ↓p-Akt	Inhibit the cell viability and promote apoptosis	Li et al. (2019)
	Non-small cell lung cancer	Suppressor gene	Downregulation	↑PKM2, ↑p-Akt	Confer drug resistance	Chen et al. (2020)
	Melanoma	Oncogene		↑p-Akt, ↑MMP2, ↑MMP9, ↑Slug, ↓E-cadherin	Promote proliferation and invasion	Zhu et al. (2018)
	Melanoma	Oncogene	Downregulation	↓p-PI3K, ↓p-Akt	Anti-cancer	Bi et al. (2019)
	Hodgkin’s lymphoma	Oncogene	Overexpression	↑Akt	Promote proliferation	Wang et al. (2019b)
	Choriocyrarcinoma	Oncogene	Knockout	↑PI3K, ↑Akt, ↑mTOR	Reduce drug resistance	Yu et al. (2019)
Canonical Wnt/β-catenin	Pancreatic cancer	Oncogenic	Knockdown	↑p-β-catenin, ↓p-LRP6, ↓Snail	Modulate cell proliferation and migration	Sun et al. (2019)
	Colorectal cancer	Oncogenic	Overexpression	↑progranulin, ↑β-catenin, ↑c-Myc, ↑cyclin D1	Promote EMT	Ding et al. (2018a)
	Colorectal cancer	Oncogenic	Knockdown	↓β-catenin	Restore the sensitivity of drug resistance	Wu et al. (2017a)
	Breast cancer	Oncogenic	Overexpression	↑YWHAZ, ↑Slug, ↑Snail, ↑Vimentin, ↑Cyclin D1, ↑c-Myc, ↑β-catenin, ↓E-cadherin	Promote EMT	Yan et al. (2020)
	Breast cancer	Oncogenic	Overexpression	↑Oestrogen receptor activated Wnt signalling	Increase the ability of self-renewing	Wang et al. (2019a)
	Glioma	Oncogenic	Knockdown	↑GSK-3β, ↓DVL2, ↓cyclin D1, ↓β-catenin	Inhibit proliferation, invasion, migration, induced apoptosis	Guan et al. (2019)
	Glioma	Oncogenic	Knockdown	↓Vascular endothelial growth factor A, ↓MMP9, ↓Wnt5a, ↓β-catenin	Inhibit proliferation, migration, angiogenesis	Zhou et al. (2020)
	Bladder cancer	Oncogenic	Overexpression	↑EZH2, ↑Nkd1, ↓E-cadherin	Increase metastasis	Luo et al. (2013)
Canonical NF-κB	Gastric cancer	Oncogenic	Overexpression	↑p65, ↓IκBα	Promote proliferation, migration, invasion	Zhang et al. (2019b)
	Thyroid cancer	Suppressor gene	Overexpression	↓p-PI3K, ↓p-↓Akt, ↓p65, ↓p- IκBα	Inhibit viability, migration, invasion	Wang et al. (2017)
	Melanoma	Oncogene	Downregulation	↑IκBα, ↓p65, ↓p50, ↓p-PI3K, ↓p-Akt	Inhibit migration and invasion	Liao et al. (2018)
	Osteosarcoma	Oncogene	Downregulation	↑IκBα, ↓p-PI3K, ↓p-Akt	Inhibit migration, invasion	Zhao and Ma, (2018)
	Multiple myeloma	Oncogene	Knockdown	↓p65, ↓p- IκBα	Inhibit growth	Sun et al. (2017)
MAPK	Hepatocellular carcinoma	Oncogene	Overexpression	↑MAPK1	Promote aggressiveness	Ye et al. (2020)
	Hepatocellular carcinoma	Oncogene	Downregulation	Oxidative ↑stress, ↓MAPK, ↓ERK	reverse drug resistance	Ding et al. (2018b)
	Colorectal cancer	Oncogene	Overexpression	↑Ras, ↑p- Raf, ↑p-ERK, ↑p-MEK	Promote migration, invasion	Yang et al. (2018)
	Colorectal cancer	Oncogene	Overexpression	↑Raf-1	Promote metastasis	Zhang et al. (2020)
	Non-small cell lung cancer	Oncogene	Overexpression	↑ERK1/2	Increase migration, invasion	Wang et al. (2016)
	Oral cancer	Oncogene	Knockdown	↓ERK, ↓p-ERK, ↓p-p38, ↓JNK	Inhibit proliferation, migration, glycolysis	Yang et al. (2021a)
JAK/STAT	Pancreatic cancer	Oncogene	Overexpression	↑STAT3, ↓SCOS5	Promote EMT, stemness	Wang et al. (2020a)
	Non-small cell lung cancer	Oncogene	Overexpression	↓STAT3	Promote cancer development	Huang et al. (2018)
Apoptosis	Gastric cancer	Oncogene	Overexpression	↓FADD, ↓caspase-3, ↓caspase-8	Promote proliferation, inhibit apoptosis	Yan et al. (2017)
	Hepatocellular carcinoma	Oncogene	Overexpression	↓FADD, ↓caspase-3, ↓caspase-8	Promote growth, inhibit apoptosis	Ge et al., 2019a; Ge et al., 2019b
	Non–small cell lung cancer	Oncogene	Overexpression	↑Bcl-2, ↓p53, ↓Bax	Promote cancer progression and development	Zheng et al. (2019)
	Cholangiocarcinoma	Oncogene	Overexpression	↑Bcl-2	Promote proliferation, migration, invasion	Yu et al.(2020)

Although the association of H19 with various oncogenic signaling pathways in the processes of tumorigenesis and carcinogenesis was discussed in this review, certain deficiencies and problems remain, and further studies are needed to strengthen the link. Firstly, as mentioned above, one of the functions of H19 is to regulate epigenetic modifications (Alipoor et al., 2020). In contrast to the functions of sponging miRNA and interacting proteins, only a few studies have focused on the association of H19 with epigenetic regulation. Further studies are needed to determine the role of H19 in epigenetic modifications in regulating the above oncogenic signaling pathways. Secondly, in addition to the above pathways, further studies should focus on H19 participating in cancer initiation and progression by acting on other pathways, such as the p53, Hippo, Sonic Hedgehog (Shh), and PTEN signaling pathways. Thirdly, increasing evidence has shown that H19 plays an important role in various cancers *in vivo* and *in vitro*; nevertheless, clinical trials on H19 in cancer are lacking, and it is necessary to conduct more clinical trials. Additionally, the combination of basic research on H19 and clinical trials may be more proficient for the diagnosis, prevention, treatment and prognosis of various cancers. Fourth, H19 plays dual roles in cancer. In thyroid cancer, H19 exerts an oncogenic function to modulate cell viability and apoptosis by regulating the PI3K/Akt pathway, while another study demonstrated that H19, as a suppressor gene, inhibited cell viability, migration, and invasion *via* insulin receptor substrate I-mediated PI3K/Akt and NF-κB pathways in thyroid cancer cells (Wang et al., 2017; Li et al., 2019). The reason why H19 shows discrepancy results needs to be further investigated. Lastly, increasing evidence had shown that circulating H19 could as a biomarker for monitoring cancer progression (Wang et al., 2018; Zhong et al., 2020), however, the detail mechanisms are not clear in various types of cancer. Whether the regulatory mechanism of circulating H19 in cancer is the same as H19, which needs to be further investigated in the future.

## Glossary

Akt: protein kinase B
BeCSCs: breast cancer stem‐like cells
CAFs: cancer-associated fibroblasts
CRC: colorectal cancer
CREB: cAMP-response element binding protein
ceRNA: competitive endogenous RNA
EGFR: epidermal growth factor receptor
ERK: extracellular regulated protein kinases
EZH2: enhancer of zeste homolog two
FADD: fas-associating protein with a novel death domain
GSK-3β: glycogen synthase kinase 3β
HCC: hepatocellular carcinoma
HP: *Helicobacter pylori*
H19: long noncoding RNA H19
ICR: imprinting control region
IGF2: insulin-like growth factor 2
lncRNA: long ncRNA
IL: interleukin
JAK: the janus kinase
JNK: c-jun n-terminal kinase
LPR: lipoprotein receptor‐related proteins
MAPK: mitogen-activated protein kinase
MM: multiple myeloma
MMP: matrix metalloproteinases
mTOR: mechanistic target of rapamycin
ncRNA: noncoding RNA
NF-κB: nuclear factor kappa B
NSCLC: non-small cell lung cancer
PI3K: phosphatidylinositol 3-kinase
PFTK1: cyclin‐dependent kinase 14
RUNX1: runt domain transcription Factor 1
SOCS: suppressors of cytokine signaling
STAT: signal transducer and activator of the transcription
TCGA: The Cancer Genome Atlas
TNBC: triple-negative breast cancer
VGF: neurosecretory protein
YWHAZ: tyrosine 3-monooxygenase/tryptophan 5-monooxygenase activation protein zeta
ZEB1: zinc finger E-box-binding homeobox one
